# Digital Rehabilitation after Knee Arthroplasty: A Multi-Center Prospective Longitudinal Cohort Study

**DOI:** 10.3390/jpm13050824

**Published:** 2023-05-13

**Authors:** Julien Lebleu, Andries Pauwels, Philippe Anract, Sébastien Parratte, Philippe Van Overschelde, Stefaan Van Onsem

**Affiliations:** 1 moveUP, Cantersteen 47, 1000 Brussels, Belgium; 2 Service de Chirurgie Orthopédique, Hopital Cochin, 75679 Paris, France; 3 International Knee and Joint Centre, Abu Dhabi 46705, United Arab Emirates; 4 Locomotion Institute, Aix Marseille University, 13009 Marseille, France; 5 Hip and Knee Clinic, 9830 Gent, Belgium; 6 Orthopaedics Department, AZ Alma Eeklo, Ringlaan 15, 9900 Eeklo, Belgium; 7 Department of Human Structure and Repair, Ghent University, 9000 Gent, Belgium

**Keywords:** total knee arthroplasty, knee, digital rehabilitation, telerehabilitation, mhealth, individualized

## Abstract

Rehabilitation for total knee replacement (TKA) often involves in-person therapy sessions, which can be time consuming and costly. Digital rehabilitation has the potential to address these limitations, but most of these systems offer standardized protocols without considering the patient’s pain, participation, and speed of recovery. Furthermore, most digital systems lack human support in case of need. The aim of this study was to investigate the engagement, safety, and clinical effectiveness of a personalized and adaptative app-based human-supported digital monitoring and rehabilitation program. In this prospective multi-center longitudinal cohort study, 127 patients were included. Undesired events were managed through a smart alert system. Doctors were triggered when there was a suspicion of problems. The drop-out rate, complications and readmissions, PROMS, and satisfaction were collected through the app. There was only 2% readmission. Doctor actions through the platform potentially avoided 57 consultations (85% of alerts). The adherence to the program was 77%, and 89% of the patients would recommend the use of the program. Personalized human-backed-up digital solutions can help to improve the rehabilitation journey of patients after TKA, lower healthcare-related costs by lowering the complication and readmission rate, and improve patient reported outcomes.

## 1. Introduction

Total knee arthroplasty (TKA) is a commonly performed procedure to relieve pain and improve function in individuals with degenerative knee joint disorders [1]. Not only the number of procedures but also the cost associated with these surgeries is rising, making it important to find ways to monitor the postoperative trajectory and complications effectively [2].

Rehabilitation is a crucial component of postoperative care, as it can decrease pain, and improve function and activities of daily living [3]. However, traditional rehabilitation for total knee replacement patients often involves in-person therapy sessions, which can be time consuming and costly. In addition, adherence to home exercise therapy is often low, which can lead to suboptimal outcomes and increased healthcare costs [4,5]. The lack of access to rehabilitation services in remote and underserved areas can also be a barrier to effective rehabilitation [6].

Digital rehabilitation has the potential to address the limitations of traditional rehabilitation. Mobile apps and other technology-based tools provide individuals with access to rehabilitation therapy outside of traditional settings, which can increase engagement and improve adherence to therapy [7]. Digital rehabilitation also has the potential to increase access to rehabilitation services [8]. In addition, digital rehabilitation can provide real-time data and feedback on therapy progress, which can improve outcomes and enhance the overall rehabilitation experience. While the heterogeneity between digital rehabilitation systems and the lack of clear evidence regarding their effectiveness and safety need to be considered, digital rehabilitation holds great promise as a solution to enhance rehabilitation outcomes for total knee replacement patients [9,10,11]. The latest evidence on digital rehabilitation has demonstrated that it is non-inferior to face-to-face interventions and has the potential to improve outcomes for patients [12]. The growing interest in digital rehabilitation is reflected in multiple studies on telemedicine and arthroplasty in the last 5 years. This is especially true after the pandemic, as patients are looking for alternatives to traditional rehabilitation methods. Most of these systems are limited because they offer the same protocols for every patient without considering the patient’s pain, participation, and speed of recovery. Furthermore, most of the available systems are not backed up by human support in case of need.

Understanding the potential of digital rehabilitation better might help to enhance the development of personalized effective rehabilitation programs for patients undergoing knee replacement surgery, and consecutively optimize patients’ episode of care and outcomes. The aim of this study was to investigate the engagement, safety, clinical effectiveness, and satisfaction of a personalized and adaptative app-based human-backed-up digital monitoring, pain management, and rehabilitation program after knee replacement arthroplasty.

## 2. Materials and Methods

This interventional, multi-center, single-arm, prospective study was performed on 127 individuals with degenerative knee pain who utilized digital rehabilitation following TKA between January 2021 and May 2022. These patients underwent no face-to-face rehabilitation. Subjects were included in 1 French and 13 Belgian hospitals. Characteristics of participants are displayed in Table 1.

Individuals were invited to download an app-based telerehabilitation system.

The application ‘moveUP Therapy’ (moveUP®, Bruxelles, Belgium) is registered as a medical device and uses a smart virtual platform for digital rehabilitation based on objective and subjective patient data, combined with personalized interaction between a therapist and the patient. The treatment is continuously adapted and personalized automatically and clinically according to the patient’s needs.

The exclusion criteria were as follows: having any preoperative (e.g., epidural catheter, urethral catheter, intra-articular catheter) or postoperative procedure that might interfere with the rehabilitation during and after hospitalization; or having any significant medical condition (e.g., Parkinson’s disease, multiple sclerosis, cerebral vascular accident) or psychiatric disorder (active alcohol/drug abuse, etc.) that might interfere with the rehabilitation.

The intervention was a home-based digital intervention of exercise and education. Patients were monitored remotely by a physical therapist through a secured chat messaging system. The system is composed of a mobile app for the patient and a web-based portal that allows the physical therapist to look at patient data (physical activity, pain levels, medication use, exercise adherence, PROMS, pictures, videos) daily and to personalize the protocol accordingly. Objective data was collected throughout the recovery using a commercial activity tracker (Garmin Vivofit 4) worn 24/7 by the patients.

Based on the patient objective and subjective daily feedback, exercises were delivered daily, and patient-reported adherence was assessed for each of them, allowing the calculation of an adherence rate (ratio of exercises achieved/exercises given). The patient was asked to answer questions daily, and to record video of their knee range of motion weekly from the day of surgery until two months after surgery. If needed, the patients were also able to take pictures of the wound or the leg and share them through the app for appropriate adaptation of the treatment plan without any systematic physical consultation if not needed based on the picture analysis.

The educational component was delivered through educational articles at specific time points of the treatment. Pain management strategies such as activities or medication counseling were personalized based on the pain and activity data of the patient.

Undesired events were managed through a smart alert system. Doctors were triggered in case of suspicion of problems. The problems were raised directly by the patients, via data-based alerts, or by the physical therapist supervising the patient’s status daily.

The ethics committee of the Universitair Ziekenhuis Antwerpen approved the study protocol, and each patient provided written informed consent to the use of their anonymized data for scientific use.

### 2.1. Outcomes

The drop-out rate and their reasons, complications, unplanned consultations, and readmissions were collected through the app.

Active knee range of motion was assessed by the physical therapist based on videos sent by the patients. Patients were requested to lie on a flat surface and bend the affected knee as far as possible by sliding the foot towards the buttocks, without forcing. It is important to note that there is a systematic error of almost 10° compared with range of motion measured by a clinician with a goniometer [13].

Patient-reported outcomes such as the Oxford Knee Score, Knee Osteoarthritis Outcome score (KOOS), and EuroQol 5-Dimension (EQ5D) were measured before surgery and 6 weeks, 3 months, and 6 months after surgery through the app. Satisfaction was assessed using the Knee Society Score (KSS) satisfaction scale. Furthermore, a binary satisfaction question was asked: “Would you chose digital rehabilitation again?”

The quality-adjusted life year (QALY) is a health outcome measure of disease burden that combines quality and length of life [14]. One year in perfect health equals 1 QALY, and 0 represents death. To calculate the QALY gain after surgery for our cohort, we used the EuroQol 5-Dimension (EQ-5D) and the Belgian value set [15].

### 2.2. Cost–Consequence Analysis

A cost–consequence analysis approach was chosen as no comparative analysis was possible given the absence of a control group. Cost–consequence analysis is considered a logical first step towards a formal economic evaluation [16,17,18]. Direct costs were analyzed separately from outcomes. Indirect cost/benefits for patients (travel time reduction) were not considered.

The per-person cost of the digital intervention was calculated from a healthcare perspective (medical costs), multiplying time logged by the care providers by their hourly salary in Belgium.

The savings of the digital intervention were estimated by assuming that alerts solved digitally prevented medical consultation with a general practitioner, or even readmission.

### 2.3. Statistics

The impact of the intervention was assessed by determining the change between the preoperative and the 6 months timepoints. Analysis of differences in the patient-reported outcomes was performed using an independent samples *t*-test or the Mann–Whitney U test. A significance level of 0.05 was used.

## 3. Results

### 3.1. Participants

**Figure 1:**
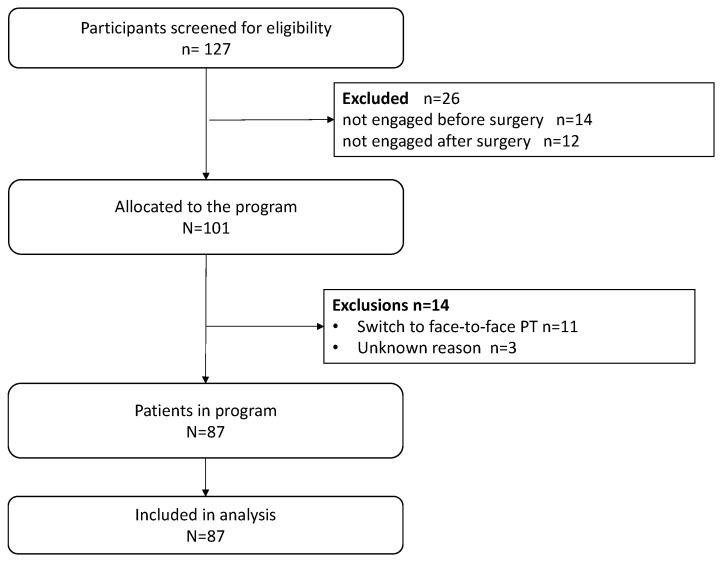
Flow diagram.

**Figure 2:**
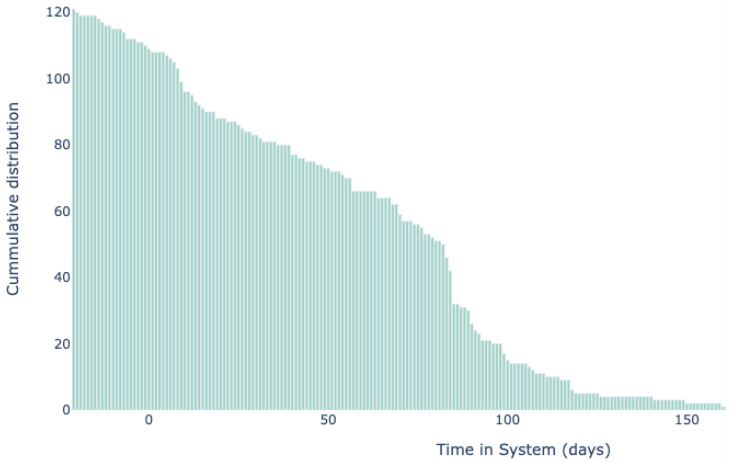
Use of digital rehabilitation.

A total of 127 patients who were referred to the digital rehabilitation trajectory were analyzed (Figure 1). Fourteen patients did not start the trajectory, and twelve did not continue after surgery. These patients did not use any features of the digital rehabilitation and were thus excluded from further analysis. Fourteen patients dropped out of the digital program for the following reasons: preference for in-person physiotherapy (*n* = 11), unknown (*n* = 3). The system was used for an average of 83 days. Patients’ use of the system is shown on the graph in Figure 2. Average adherence with the exercise achievement was 77% over the whole rehabilitation journey. A lower adherence was observed during the first two weeks after surgery (Table 2).

### 3.2. Adverse Events

Two patients were readmitted to the hospital for manipulation under anesthesia corresponding to a readmission rate of 2.4%.

**Figure 3:**
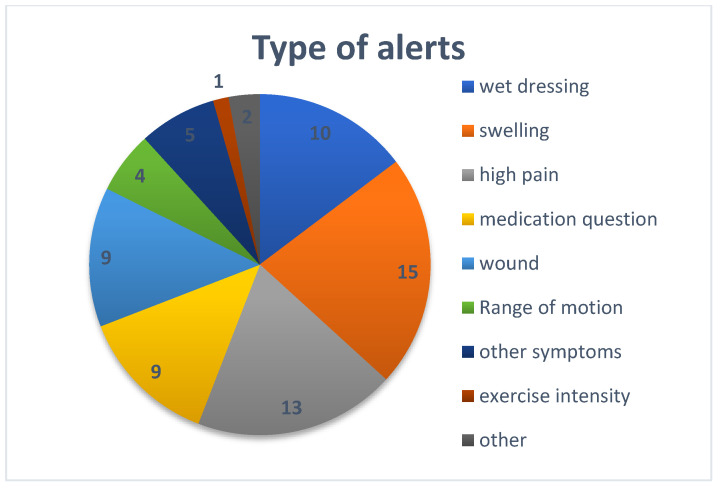
Type of alerts raised by the patient and the physical therapists.

A total of 67 alerts were raised through the web-based platforms for 32 patients (39% of the patients). A total of 32 alerts were raised by the patients (48%), and 35 by the physical therapist (52%). The types of alerts are displayed in Figure 3.

The three most frequent actions were medication change (27%) and wound information and reassurance and referral (15%). The details of doctors actions are displayed in Table 3.. Ten physical consultations were generated by the referrals. Doctor actions through the platform potentially avoided 57 consultations (85% of alerts).

### 3.3. Outcomes

The median active range of motion at 6 weeks post-operation was 105° (SD: 19°).

Significant improvements were seen at 6 months in all KOOS subscales (Table 4), amounting to 14 points in KOOS-Pain, 20 points in KOOS-Symptoms, 22 points in KOOS-Function, and 26 points in KOOS-QoL. The mean QALY gain (measured with the EQ-5D questionnaire) was 0.26 (0.25).

To the question: “Would you choose digital rehabilitation again?”, 89% of the patients answered yes.

### 3.4. Cost–Consequence Analysis

The cost of the digital intervention was EUR 257.5 per person.

This cost comprised the activity tracker (Garmin Vivofit) of EUR 60, an intake cost of EUR 22.5 (15 min by customer support and 15 min by physical therapist), and the average cost of physical therapy follow-up of EUR 170 (average of 18 min per week per patient, standard deviation of 5 min) and medical follow up of EUR 5 (67 interventions of 4 min on average, spread over 86 patients).

Intake costs included onboarding, explanations, and technical support. Intake cost consisted of 15 min remote interactions before surgery with technical support for each participant.

**Figure 4:**
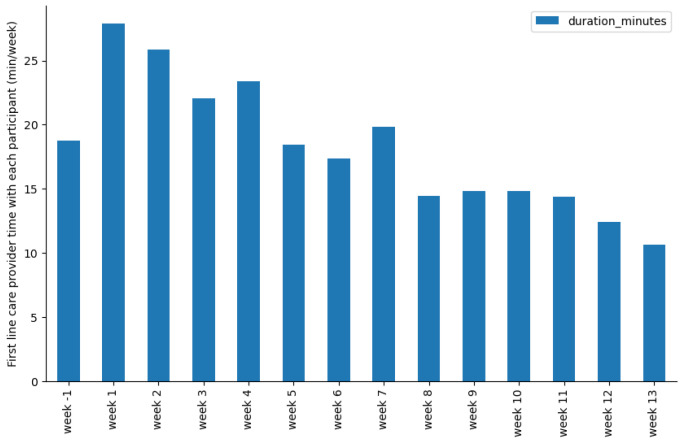
First-line care provider time spent with each participant on a weekly basis over the whole rehabilitation period.

Follow-up costs were calculated from logs that care providers completed during the study. The physical therapists (first-line care provider) logged an average of 18 min per week (SD = 5 min) with each participant (Figure 4). The doctors (second-line care provider) logged 67 instances of solving alerts, with an average of 4 min (SD = 2 min). The time spent by care providers is low because of asynchronous communication, escalation process, and platform efficiency.

The hourly salary in Belgium at the time of the study was 25 EUR/h for technical and customer support, 40 EUR/h for physical therapists, and 100 EUR/h for medical doctors.

The total direct savings of the digital intervention consisted of two aspects: reduced physical therapy costs and reduced unplanned consultations.

Traditional physical therapy after total knee replacement consists of 25 (France) to 41 (Belgium) physical therapy sessions, for an amount of EUR 500 to 1148.

Consultations with a medical doctor cost EUR 27 in Belgium. A total of 57 alerts were digitally solved for 87 patients. This represents a potential amount of EUR 1539 saved, 18 EUR/patient.

Therefore, the total direct saving potential of the digital rehabilitation solution ranges from EUR 252.5 (France) to 900.5 (Belgium).

## 4. Discussion

The aim of this study was to investigate the engagement, safety, clinical effectiveness, and satisfaction of a personalized and adaptative app-based digital monitoring and rehabilitation program after knee replacement arthroplasty. A high adherence was found, with an average use of 83 days. The complication rate was low, and undesired events were managed remotely in 85% of cases, avoiding unnecessary face-to-face consultations. Clinical scores improved significantly, and most of the patients would choose digital rehabilitation again and recommend it to other patients.

One of the limitations of the study was the absence of a control group (traditional face-to-face rehabilitation program), and the results of this study cannot be compared directly with the results of standard rehabilitation programs. The goal of the study was, however, not to show the superiority or the non-inferiority of this type of program, but to examine the feasibility, the adherence, and the safety regarding the management of the complications and the functional results. Additionally, it was found that 11% of patients stopped the program soon after surgery, mostly due to a lack of physical contact with their physical therapist or because they wanted and needed to preserve a pre-existing relationship with their physical therapist. It is important to highlight the fact that there was no reimbursement difference between the digital care program and a standard in-person physical care program in the study settings (France and Belgium). Therefore, the cost was not a reason to explain the attrition rate, and this outlines the importance of finding ways to reduce it in the future [19].

The attrition rate was low compared to previous studies, reporting between a 7% and 45% attrition rate [12,20]. The results of our study demonstrated a very high adherence level to the proposed exercises (77%). The current literature provides very little evidence surrounding patient adherence to exercise recommendations after TKA, which may also impact the implementation of these interventions [21]. A huge variability exists concerning the type of exercises and the adhesion during the in-person physical therapy programs, except for very-well-designed research studies on the impact of physiotherapy programs after TKA [22]. Therefore, very little data are reported for classic in-person programs during standard clinical practice [5]. Using a mobile application might be ideal to standardize the follow-up of PT interventions after TKA and provide direct quantitative feedback to the patients, which increases their motivation [7]. Usually, exercise adherence decreases over time [16,23], while in our study, it stayed high until the end of the rehabilitation program. It is likely that the closed feedback provided by the data and the human intervention provided to the patients by the physical therapists daily through the app increased their motivation [7,24].

With the global rise in TKA procedures, optimizing postoperative management to enhance patient-reported outcomes is of paramount importance, especially when bundle payment models apply [25]. Shorter hospitalization stays after TKA [26,27] present a need for safer and more personalized postoperative rehabilitation protocols. The most common severe complications which generate important costs are infection, deep vein thrombosis, and manipulation under anesthesia [28,29]. As a result of procedure-related complications, the average 90-day readmission rates vary widely in the literature, ranging from 3.5% to 15.6% in TKA [30,31]. The complication rate in our study was only 2.4% (two readmissions), for manipulations under anesthesia. This low rate might be attributed to the smart alert system that triggered the concerned care provider. In our study, frequent patient concerns regarding wound healing, pain, and medication were frequently observed, requiring attention from the healthcare professionals through the app. Indeed, 39% of the patients expressed concerns, but the ability to seek answers through the mobile application helped to reduce the number of outpatient consultations to only 10. These early referrals through the app with an immediate medical response might have been a factor in preventing more serious complications. These results highlight the potential benefits of implementing digital solutions in healthcare to streamline communication between patients and healthcare professionals, thereby reducing healthcare costs through the reduction of unnecessary consultations while limiting the rate of complications.

The results of our study demonstrated a significant improvement in the Oxford Knee Score, all KOOS sub-scores, and the KSS satisfaction sub-score at 6 months post-surgery. The significant improvements were similar to those previously reported after in-person interventions in Belgium and The Netherlands [32,33,34], and the population studied was representative of the registry in terms of demographics [35]. The absence of direct comparison with standard protocols in our study limits further conclusions, but the results of our study confirmed previous reports in the literature. In a study by Hardwick-Morris et al., comparing digital rehabilitation to conventional rehabilitation, it was shown that there was no significant difference, after 12 months, in any KOOS or KOOS, JR scores [18]. The study conducted by Timmers et al. demonstrated that the implementation of a digital application post total knee arthroplasty can lead to a significant reduction in daily pain levels and improvement in functional outcomes [20]. Additionally, digital interventions implemented following joint arthroplasty have been found to enhance patient adherence and postoperative satisfaction, making it a potential cornerstone for new pre- and postoperative care pathways in arthroplasty [36]. Based on the results in the literature, it could be said that digital rehabilitation is at least non-inferior to conventional rehab [12].

Introducing new technologies into clinical practice requires careful consideration of patient acceptance and ease of use. In our study, we assessed patient satisfaction with the system and found that the patient promoter/satisfaction score was 89%, indicating a high level of patient acceptance. This finding is consistent with a study by Correia et al., which reported a 90% satisfaction rate [37], or with the study by Scheper et al., which reported high usefulness perceived by patients using an app to specifically monitor wound problems [38]. The convenience of these systems for patients and their caregivers appears to overcome the challenges associated with implementing new technology.

Previous studies have already indicated that digital rehabilitation is an economically viable alternative to traditional in-person care for post knee arthroplasty rehabilitation [39,40]. The biggest difference with previous studies lies in the asynchronous design of the digital rehabilitation used in this study, which reduced the cost drastically. Part of the savings can be used by the healthcare payer to fund the digital platform, so it leads to no costs for the healthcare providers. Furthermore, apart from the cost factor, implementing an asynchronous approach for delivering physical therapy interventions may also alleviate the time constraints imposed on therapists [40], allowing them to dedicate more time to individualizing treatment plans and offering personalized attention to patients with higher needs. More detailed comparison with other studies is difficult, as our follow-up time was relatively limited compared to usual cost studies with a long-term view up to 5 years [14,41]. Indirect costs such as travel costs were not considered. In Belgium, these costs are not covered by insurance. It is likely that the travel reduction participated in the high satisfaction rate of participants.

## 5. Conclusions

In a cohort of patients following a digital rehabilitation program after knee arthroplasty, engagement was high and undesired events were carefully managed through a smart alert system which avoided unnecessary consultations. Clinical improvements were similar to those in other studies, reinforcing the latest review, who stated that digital rehabilitation is non-inferior to conventional rehabilitation. Digital rehabilitation solutions can help to make rehabilitation accessible to everyone and to lower healthcare-related costs by lowering the complication and readmission rate and the rate of unnecessary consultations.

The personalized adaptative human-backed-up app-based digital rehabilitation program for TKA patients presented in this study has a high patient promotor score (satisfaction), as well as a high adherence score (engagement), which make it an ideal rehabilitation partner as it provides good care to the patients (safe and effective) and provides unseen data feedback to the healthcare providers.

## Figures and Tables

**Table 1:** Characteristics of study participants.

Characteristic	Cohort (*n* = 127)
Age (years), mean (SD)	62 (9)
Gender (%)	
*Female*	51
*Male*	49
BMI, mean (SD)	31 (5)

SD = standard deviation.

**Table 2:** Patient engagement over time.

	Average	W1-2	W3-4	W5-6	W7-8	W9-10	W11-12
Exercises performed (%)	77	67	85	85	77	77	70
Daily questionnaire filled (%)	80	76	89	88	85	82	77
Assessment video performed (%)	71	93	86	75	76	/	/

W = week post-surgery, /: no standard assessment requested.

**Table 3:** Doctor actions in reaction to alerts.

Category	Frequency n (%)
Medication change	18 (27)
Medication info and reassure	8 (12)
Wound care	6 (9)
Wound info and reassure	10 (15)
Symptoms info and reassure	12 (17)
Referral	10 (15)
Other	3 (4)

**Table 4:** Patient reported outcomes.

	Preop		3 Months		6 Months		6 m/Preop Difference	
	Mean	SD	Mean	SD	Mean	SD	Mean	SD
Oxford Knee	24	8	33	8	38	7	14	9
KOOS Symptoms	51	18	61	18	70	19	20	21
KOOS Pain	44	19	67	20	78	19	36	20
KOOS ADL	49	20	71	21	78	18	31	22
KOOS QoL	30	18	50	20	56	22	26	27
KSS Satisfaction	15	7	25	8	30	8	16	11
QALY	0.59	0.26	0.77	0.22	0.85	0.12	0.26	0.25

SD = Standard deviation, KOOS: Knee Injury and Osteoarthritis Score, ADL: Activities of Daily Living, QoL: Quality of Life, QALY: Quality-Adjusted Life Years.

## Data Availability

The data presented in this study are available on request from the corresponding author. The data are not publicly available due to privacy reasons.
